# Letter from J. E. Snodgrass, M. D. to the Baltimore Editor

**Published:** 1841-09

**Authors:** J. E. Snodgrass


### ARTICLE X.

*Letter from* J. E. SNODGRASS, M. D. *to the Baltimore Editor.*

DEAR DOCTOR:— *Baltimore, June* 2, 1841.

YOU recollect that when I last had the pleasure of seeing you, our conversation turned upon dental irritation as concerned in the production of fatal diseases. I happened to allude to the painful and sudden death of my father as an example of the powerful, yet hidden influences of dental affections, as a case in point. Your request, that I would report the case, I now attempt to comply with, although I confess I do so with some reluctance. In the first place, because it is a painful reminiscence; and, secondly, not having been at all *indoctrinated* at the time of its occurrence, I fear that the partial report I shall make, will prevent the case from being so valuable to you as you were disposed to consider it. In order to render facts relating to any branch of remedial science, fully useful, they should, if possible, be reported not only honestly, but professionally. Besides, one can hardly be expected to bear facts in memory for a period of ten years, (which have elapsed since the occurrence under notice,) so as to give an authentic account of them. However, there could be no circumstances of life, so likely to make an indelible impression, as that of a father called, with a few hours warning, to a death by the most horrific of all diseases—*phrenitis.* I will state the essential facts, then, as briefly as possible. At the time of his death his age was about fifty-two. His general health had been good, with the exception of occasional attacks of urinary irritation accompanied with sedimentitious secretions and difficulty of retention. This affection was ascribed to the influences of a gouty diathesis, which he left as an unwelcome legacy to his only son. Some months before his death he contracted a severe cold, which resulted in a protracted indisposition, the nature of which I do not recollect. When able to leave his room and resume the supervision of his business, he imprudently walked out after a rain, in a pair of very thin soaled slippers; and getting his feet wet in that way, a relapse was the result. The relapse was accompanied by a general odontalgic affection, in which a number of his teeth participated, (they being, generally, very much decayed and fractured, from a neglect far too general, in retired districts especially,) to the production of much nervousness of temper and feebleness. The inflammatory action increased gradually, until the entire inferior maxilary bone, (of the left side, I think,) appeared to have become involved in it. It had, now, arrived at that state which is styled *jaw-ache*, in common parlance. A few days previous to his death the inflammatory action reached the ear, and induced what he described as a deep-seated benumbed pain, accompanied by a disagreeable swimming in the head, and drumming in the ears. Yet he was, at no time, bed-fast on account of his indisposition. On the evening before his death we supped together as usual, he conversing with ordinary freedom. About nine o'clock at night I was awakened, and informed that he was "crazy." I hastened to his chamber, only to discover him completely frantic. Delirium, which is commonly an early companion of phrenitis, had already supervened, and displayed its presence by the most terrific symptoms. His eyes were turgid, his face deeply flushed—his countenance wild and maniacal. His vision and sense of hearing appeared entirely gone, so that he did not seem to recognize me. Nor did he respond to my queries pronounced, with my mouth placed over the ear, in the loudest tones. Incoherent mutterings were all that he uttered, while he tossed to and fro with a muscular strength which required the assistance of the most athletic man-servant to keep him any thing like quiet. He continued in this state for several hours, and then sank into a somewhat soporific state, characterized by what I have since recognized as coma. In this comatose state he died about ten o'clock the following morning, despite of blood-letting and other active remedies applied as promptly as possible.

I have thus been particular in my detail of the symptoms that you may recognize in the case, the presence of the disease to which I have already ascribed my father's sudden death. I have not the least doubt, that odontalgia was the indirect cause of the suddenness of his mortality. The ear-ache, doubtless, resulted *sympathetically* from the tooth-ache; while the phrenitis was the *finale*—taking place under the control of an insiduous inflammation, whose progress, perhaps, his medical attendant little suspected, if at all. If there could be any difficulty in tracing the first steps of the inflammatory action, there surely could be none in accounting for the appearance of phrenitis, after the appendages of the brain became involved ever so slightly—for medical writers mention cases the result of phlegmonous swellings about the scalp, and of small-pox, and erysipelas of the head and face. The main questions to be determined, then, are the modes in which the inflammation originally seated in the teeth, was conducted to the auditory apparatus, and, next to the brain and its coverings. Now, to say nothing about the sympathy which parts of the animal economy less delicately organized, manifest for each other—it is clear to the most superficial medical observer, that the muscular tendinous, and vascular connections between the jaw and the ear, and between the ear and the brain, are exceedingly intimate. But, if we had any doubts on this score, the *nervous* connections between these parts need merely be alluded to, in order to render the inference clear, that phrenitis and sudden death, were the results of a simple odontalgia. The nerves distributed to the teeth and ear, have a common origin in branches of the *fifth pair*—while the immediate nervous relation of the auditory apparatus, with the cerebral mass, and its great covering, the dura mater, is strikingly apparent.

I do not deem any further comments pertinent from me; for, if the influences of simple odontalgia *did* produce the catastrophe I have ascribed to it, the case will speak for itself, in tones which ought to awaken the community to a sense of the perils incurred by what they are in the habit of styling "a mere tooth-ache." But I cannot suffer the present opportunity to pass, as much as I am hurried, without thanking you and your colleagues, (the worth of the teachings of one of whom I have experienced) in the name of humanity for the good you are effecting through your lectures in the COLLEGE OF DENTISTRY. Your recent work has my fullest approbation—although calculated to make me feel quite ignorant of an art I am sometimes called upon to practise, and used to be *compelled* to practise when resident in the country. It contains a great mass of valuable statistics, and bears the impress of theories built upon the true Baconian basis.

If I were disposed to be jealous, I would say that the members of your art are making daring inroads upon the provinces of medicine and surgery. Whether the more selfish and narrow-souled of my profession are pleased, or displeased, is not the main matter; for, I apprehend, the more enlightened classes of the people have discovered, that what is our loss, is their gain. The time is coming when the *forceps* will be talked of as *the* cure for many a disease, whose eradication was supposed, hitherto, to depend upon the continuance of the "opium trade." I have no doubt in the world that many a case of *tic douloureux*, which has consumed much opium, and expended hundreds of hard-earned dollars, (and been subjected to a painful operation for division of nerves at last) might have been forever suppressed by the simple operation of lifting a useless tooth from its fœtid repose. Many of the nervous headaches we hear of, I am inclined to believe—nay, I know it, are referable to dental irritation, and that they might be greatly alleviated, if not banished wholly, by a few sittings in the dental chair.

I have only one concluding remark to make. It is that, I hope the time is coming when the march of science in your branch of the profession, shall have drawn the line of demarcation between quackery and true merit, so that the people at large may be able to tell, at the first glance, the sheep from the *goats*. This, nothing will so speedily effect as a diffusion of scientific knowledge among the masses. Nothing enables a non-professional man to discriminate between the miserable pretender and the truly indoctrinated, like an outline-view of the structure and diseases of the human frame, and the remedial means applicable to their cure. It may seem paradoxical in me, but I do believe, that quackery would prevail far less, if the people at large, knew more of the secrets of the profession of medicine.

Very truly yours, J. E. SNODGRASS.

